# The Health of the City

**Published:** 1854-12

**Authors:** 


*Health of the City of Buffalo.*— As has happened in previous years, our summer visitation of cholera has been followed by a period of unusual health.

Doctors—always an unthankful race—are growling about the dearth of business, or spend their time quietly in their offices, warming themselves by the glowing grate and looking upon the chill November storms without. Occasionally one, feeling the necessity of exercise and companionship, gets under an umbrella and peregrinates among his medical friends. There long stories are told, experiences compared, and previous struggles with disease are recounted with all their "hair-breadth 'scapes." But all this does not pay. The doctor has as good a claim to pray "give us this day our daily bread" as any other. And daily bread can only come through the sufferings of others. Here is a quandary, a dilemma, a *quæstio vexata* for divines. Shall the doctor in his morning orison omit that part of his petition?

To divines we leave it. We never venture upon ethics or moral questions without coming off with a faith impaired, and a head sore puzzled about many things. H.

